# Quality of life among patients with chronic non-communicable diseases during COVID-19 pandemic in Southern Ethiopia: A cross-sectional analytical study

**DOI:** 10.3389/fpsyt.2022.855016

**Published:** 2022-09-21

**Authors:** Mohammed Ayalew, Bedilu Deribe, Siraj Hussen, Semira Defar, Abel Gedefaw

**Affiliations:** ^1^School of Nursing, College of Medicine and Health Sciences, Hawassa University, Hawassa, Ethiopia; ^2^School of Medical Laboratory, College of Medicine and Health Sciences, Hawassa University, Hawassa, Ethiopia; ^3^Department of Midwifery, College of Medicine and Health Sciences, Hawassa University, Hawassa, Ethiopia; ^4^Department of Obstetrics and Gynecology, School of Medicine, College of Medicine and Health Sciences, Hawassa University, Hawassa, Ethiopia

**Keywords:** COVID-19, quality of life, QOL, predictors, Ethiopia

## Abstract

**Background:**

The COVID-19 outbreak became a continuing global health agenda. It has a significant impact on individuals’ quality of life (QOL). Patients with preexisting medical conditions may have severely reduced QOL. The aim of this study was to assess QOL and its associated factors among patients with chronic non-communicable diseases (NCDs) during COVID-19 pandemic at Sidama Regional State, southern Ethiopia.

**Methods:**

We conducted a multicenter, cross-sectional study from 1 June to 1 September 2021. A total of 633 participants took part in the study, using an interviewer-administered structured questionnaire. The QOL was measured using the World Health Organization Quality of Life (WHOQOL-BREF) Scale, which has 12 items. To describe different variables, descriptive statistics were employed. To find independent factors associated with QOL, we used multivariable linear regression analysis. *P*-value of < 0.05 was declared statistically significant at 95% confidence interval (CI).

**Results:**

The majority (56.4%) of participants were male and about half (53.1%) had a diagnosis of diabetes mellitus. The multivariable linear regression model showed statistically significant negative association between different independent variables such as age (β = −0.188, 95% CI = −0.238 to −0.139), being female (β = −1.942, 95% CI = −3.237 to −0.647), duration of illness ≤ 5 years (β = −4.222, 95% CI = −6.358 to −2.087), alcohol use in the past 3 months (β = −4.574, 95% CI = −6.905 to −2.243), common mental disorder (CMD) (β = −1.512, 95% CI = −2.924 to −0.100), insomnia (β = −0.274, 95% CI = −0.380 to −0.168), and QOL. Also, there is a statistically significant positive association between QOL and being illiterate (β = 3.919, 95% CI = 1.998–5.841) and living in the rural area (β = 2.616, 95% CI = 1.242–3.990).

**Conclusion:**

In general, the findings confirmed that the COVID-19 pandemic had a negative impact on patients with chronic NCDs QOL. The QOL was significantly influenced by age, gender, educational status, residence area, duration of illness, alcohol use, CMD, and insomnia during COVID-19 pandemic. Thus, this study suggests that addressing insomnia, co-morbidities of mental disorders, and alcohol use has the potential effect to improve the QOL of patients with chronic medical illnesses.

## Introduction

The coronavirus disease (COVID-19) pandemic, which began in China in December 2019, is still posing a global health risk (1). Over 304 million confirmed cases and 5.4 million deaths had been reported globally in the 2 years since its emergence, as of the first week of January 2022 (2). As of 2 January 2022, there were over 400 thousand COVID-19 confirmed cases in Ethiopia, and the virus was responsible for about 7,000 fatalities. Risk communication and awareness campaigns are being used to address worries about the alarming rise in COVID-19 cases and the widespread dissemination of flu-like symptoms among Ethiopians. Additionally, the COVID-19 vaccine has been given in doses totaling close to 11 million up to January 2022 (3).

The severity and mortality of COVID-19 infections are thought to be increased in patients with preexisting medical conditions like diabetes mellitus (DM), hypertension, and malignancies (4). The case-fatality ratio was higher in patients with cardiovascular diseases (10.5%), diabetes (7.3%), and hypertension (6%) compared to the general population (2.3%) (5). Aside from physical health, COVID-19 has had a negative impact on mental health, causing significant anxiety and depression in people (6), and interfering with daily lives, jobs, and relationships. People are afraid of infection, dying, and losing family members during the COVID-19 outbreak. At the same time, many people have lost or are in danger of losing their jobs, have become socially isolated and separated from loved ones, and have seen stay-at-home orders implemented in some countries in drastic ways (7).

As a result, whether infected or not, COVID-19 has had a significant impact on people’s quality of life (QOL) (8, 9). The COVID-19 outbreak is regarded as an unforeseen traumatic life event that has harmed individuals’ QOL in general and particularly their health-related quality of life (HRQOL). HRQOL is a multidimensional concept that describes how an individual assesses his or her mental, physical, emotional, and social wellbeing (10). Many studies have been conducted to investigate the QOL of recovered COVID-19 cases in the general population, hospitalized patients, and chronic illness patients (9, 11–13).

The COVID-19 pandemic has a significant negative impact on QOL, especially in terms of physical, mental, social, and spiritual wellbeing (9, 14–17). A recent study in Bangladesh found a link between chronic diseases such as hypertension (HTN), diabetes mellitus (DM), heart disease, asthma, kidney diseases, and cancer and significantly lower QOL in all domains (18). According to a recent Egyptian study, 64% and 62% of diabetic patients reported poor physical and mental QOL, respectively. (19). In a similar study conducted in the Netherlands, 43% of patients with chronic kidney disease reported that the COVID19 pandemic had reduced their QOL (20).

People with non-communicable chronic diseases (NCDs) found it challenging to see a doctor and have their prescriptions filled (21) during the COVID-19 pandemic. During the peak of the COVID-19 pandemic, movement restrictions and social isolation became the new normal, contributing to a substantial reduction in people’ activities, which is associated with a significant decline in overall QOL (22). As a result, it is essential to look into how the COVID-19 pandemic affected the QOL of individuals living with chronic NCDs.

The unbearable impact of the COVID-19 pandemic may have had a significant impact on the QOL of patients with chronic medical conditions. To the best of our knowledge, data on QOL assessment among chronic disease patients in response to the COVID-19 pandemic are scarce, particularly in low-income countries like Ethiopia. Furthermore, there is a scarcity of data on the relationship between COVID-19-related psychological complications (such as depression, anxiety, and stress) and QOL among chronic disease patients during the COVID-19 pandemic. Therefore, this study fills a research gap by (1) describing QOL among patients with chronic medical conditions and (2) identifying the association between various socio-demographic, clinical, and psychological factors and QOL to identify significant predictors of QOL among patients with chronic medical conditions during the era of COVID-19 pandemic.

## Methods and materials

### Study design, area, and period

We conducted a cross-sectional study between 1 June and 1 September 2021 at four selected hospitals [Hawassa University Comprehensive Specialized Hospital (HUCSH), Adare General Hospital (AGH), Yirgalem General Hospital (YGH), and Leku Primary Hospital (LPH)] in Sidama National Regional State, southern Ethiopia.

### Study participants

This study was conducted among patients with chronic non-communicable diseases such as diabetes mellitus, hypertension, chronic cardiovascular diseases, and respiratory diseases (e.g., asthma) that have regular follow-up visits. Patients receiving follow-up care in the outpatient departments of the four hospitals were consecutively requested to participate in the study, if they met the following criteria: (I) age ≥18 years with chronic NCDs confirmed by physicians; (II) clinically stable and able to understand the purpose of the study; and (III) patients without any known psychiatric and neurocognitive disorders. However, patients with chronic NCDs who were admitted to the emergency/inpatient department for any reason were excluded from the study. We were planning to include 650 patients with chronic NCDs from the four hospitals based on the monthly patient flow (250 from HUCSH, 150 from AGH, 125 from YGH, and 125 from LPH). We used a consecutive sampling technique, and patients with chronic NCDs who visited hospitals during the study period and met the inclusion criteria were included in the study until the final study sample size was reached.

### Data collection methods

A structured self-administered questionnaire was used to gather data. The questionnaire is divided into various sections, such as the socio-demographic and clinical characteristics of the patient, the Oslo Social Support Scale (OSSS), the Self-Reporting Questionnaire-20 (SRQ-20) to evaluate common mental disorders (CMD), the Insomnia Severity Index (ISI) to evaluate insomnia, and the World Health Organization Quality of Life Instruments (WHOQOL-BREF) to evaluate QOL. The questionnaire was prepared in English and translated to the local language Amharic. The Amharic version of the questionnaire was used to collect the data.

#### Social support

The level of social support among patients with chronic NCDs was assessed using the 3-item Oslo Social Support Scale (OSSS), and the scores range from 3 to 14. It is categorized as poor [3–8], moderate [9–11], and strong [12–14] social support (23).

#### Common mental disorder

A 20-item (SRQ-20) WHO screening tool was used to assess CMDs (24). Only binary (yes/no) questions are included, with “1” indicating the presence of a symptom and “0” indicating the absence of a symptom. The SRQ-20 item questions cover depression, anxiety, and psychosomatic complaints, which are all classified as CMD (25). The SRQ-20′s validity, reliability, and cutoff score vary by population (culture, language, setting, and gender) in different settings (25–28). With a sensitivity of 78.6% and specificity of 81.5%, the SRQ-20 had good internal reliability (α = 0.78) and an optimal cutoff score of 5/6 (29). The SRQ-20 measure demonstrated good internal consistency (Cronbach’s α = 0.89) in our study.

#### Insomnia

The ISI is a 7-item self-assessment questionnaire that assesses the nature, severity, and impact of insomnia (30). The dimensions evaluated are severity of sleep onset, sleep maintenance, early morning awakening problems, sleep dissatisfaction, interference of sleep difficulties with daytime functioning, noticeability of sleep problems by others, and distress caused by the sleep difficulties in the last month. Each item is rated on a 5-point Likert scale (e.g., 0 = no problem; 4 = very severe problem), yielding a total score ranging from 0 to 28. The total score is divided into four categories, namely, no insomnia [0–7], sub-threshold insomnia [8–14], moderate insomnia [15–21], and severe insomnia [22–28]. A higher score indicates a severe insomnia (30–32). The ISI measure demonstrated very good internal consistency (Cronbach’s α = 0.96) in our study.

#### Quality of life

We used the adapted version of 12 items (9), from the WHOQOL-BREF Scale to assess the impact of the COVID-19 pandemic on QOL (33, 34). The adapted WHOQOL-BREF Scale had 12 items, each with a five-point rating from 1 = very low to 5 = very high; thus, the lowest possible score was 12, and the highest possible score was 60 for the total scale. Low scores indicate a lower QOL as a result of the COVID-19 pandemic’s negative effects. The QOL measure demonstrated good internal consistency in the previous study (Cronbach’s α = 0.81) (9). The WHOQOL-BRIEF 12 items (Supplementary File 1) used in our study demonstrated good internal consistency (Cronbach’s α = 0.82).

### Data analysis

Collected data were entered to Epi-data version 3.1 and exported to SPSS (Statistical Package for Social Sciences) version 24 for analysis. Descriptive statistics such as frequency, percentage, mean, standard deviation, and median were used to describe different variables. Assumptions such as normality, lack of multi-collinearity among explanatory variables, presence of linearity relationship, independence, and homoscedasticity of the errors were checked. Simple and multivariable linear regressions were performed to identify independent predictors of QOL. *P*-values of < 0.05 were declared statistically significant at 95% confidence interval (CI).

## Results

This study included 633 participants. The majority of study participants (56.4%) were male, and 64.8% were married. About half (55.6%) were Protestant religious followers, followed by Orthodox Christians (25.9%), and about one-fourth (27.6%) were illiterate. More than one-fifth (22.7%) worked as a farmer, while 55.8% lived in urban. The mean age of the respondents was 46.49 ± 17.71 years as described in Table 1.

**TABLE 1:** Socio-demographic characteristics of study participants at Sidama National Regional State, southern Ethiopia, 2021 (*n* = 633).

Variable	Categories	Frequency	Percentage (%)
Age (mean ± SD)	46.49 ± 17.71		
Sex	Male	357	56.4
	Female	276	43.6
Marital status	Single	135	21.3
	Married	410	64.8
	Divorced	36	5.7
	Widowed	52	8.2
Religion	Protestant	352	55.6
	Orthodox	164	25.9
	Muslim	103	16.3
	Others	14	2.2
Educational status	Illiterate	175	27.6
	Primary	162	25.6
	Secondary	131	20.7
	College and above	165	26.1
Occupation	Gov’t employee	121	19.1
	Private employee	67	10.6
	Merchant	90	14.2
	Student	68	10.7
	House wife	102	16.1
	Farmer	144	22.7
	Jobless	21	3.3
	Other	20	3.2
Place of residence	Rural	280	44.2
	Urban	353	55.8

About half of the participants (53.1%) had diabetes mellitus, followed by hypertension (17.5%), and nearly one-third (31.6%) had a comorbid diagnosis. The majority of participants (57.5%) had been sick for 5 years, and 9.6% and 7.7% had used alcohol and khat in the previous 3 months, respectively. More than one-third (35.4%) of all participants had poor social support, while about half (52.4%) had moderate social support. The mean SRQ-20 and ISI scores were 6.06 ± 5.09 and 6.62 ± 6.89, respectively (Table 2).

**TABLE 2:** Clinical characteristics of study participants at Sidama National Regional State, southern Ethiopia, 2021 (*n* = 633).

Variable	Categories	Frequency	Percentage (%)
Diagnosis	Diabetes mellitus	336	53.1
	Hypertension	111	17.5
	Asthma	49	7.7
	CVD	82	13.0
	Others*	55	8.7
Comorbid diagnosis	Yes	200	31.6
	No	433	68.4
Duration of illness	≤5 years	364	57.5
	6–10 years	210	33.2
	≥11 years	59	9.3
Alcohol Use in the past 3 months	Yes	61	9.6
	No	572	90.4
Cigarette Use in the past 3 months	Yes	17	2.7
	No	616	97.3
Khat Use in the past 3 months	Yes	49	7.7
	No	584	92.3
Social support	Poor	224	35.4
	Moderate	332	52.4
	Strong	77	12.2
Common mental disorders	Yes	209	33.0
	No	424	67.0
Insomnia	Yes	249	39.3
	No	384	60.7

CVD, Cardiovascular disorders.

*Epilepsy, stroke, neurological, renal, or hepatologic disorders.

### Quality of life of participants

According to the WHOQOL-Brief (12-items) scale score, the mean QOL score of the participants was 33.07 ± 8.90 with a minimum score of 12 and a maximum score of 53.

### Independent factors associated with quality of life

Estimates of the multivariable linear regression model showed a statistically significant and negative association between different independent variables such as age (β = −0.188, 95% CI = −0.238 to −0.139), CMD (SRQ-20 scale) (β = −1.512, 95% CI = −2.924 to −0.100), insomnia (ISI scale) (β = −0.274, 95% CI = −0.380 to −0.168), being female (β = −1.942, 95% CI = −3.237 to −0.647), duration of illness ≤5 years (β = −4.222, 95% CI = −6.358 to −2.087), alcohol use in the past 3 months (β = −4.574, 95% CI = −6.905 to −2.243), and the outcome variable QOL during COVID-19 pandemic. On the contrary, there is a statistically significant and positive association between QOL and being illiterate (β = 3.919, 95% CI = 1.998–5.841) and living in the rural area (β = 2.616, 95% CI = 1.242–3.990) (see Table 3).

**TABLE 3:** Simple and multiple linear regression for quality of life among patients with chronic medical illness during COVID-19 pandemic at southern Ethiopia, 2021 (*n* = 633).

Variables		Simple linear regression	Multiple linear regression^†^
		β (95% CI)	β (95% CI)
Age		−1.158 (−0.195, −0.121)*	−0.188(−0.238, −0.139)*
CMD (SRQ−20 scale)		−0.524 (−0.655, −0.394)*	−1.512(−2.924, −0.100)***
Insomnia (ISI scale)		−0.490 (−0.583, −0.396)*	−0.274(−0.380, −0.168)*
Sex	Male	Reference	
	Female	−0.563 (−1.964, 0.839)	−1.942(−3.237, −0.647)**
Marital status	Single	Reference	
	Married	1.958 (0.275, 3.641)***	0.654 (−1.105, 2.413)
	Divorced	−7.438 (−10.386, −4.491)*	−2.543 (−5.430, 0.345)
	Widowed	−3.729 (−6.225, −1.233)**	0.163 (−2.874, 3.200)
Educational status	Illiterate	5.302 (3.450, 7.154)*	3.919(1.998, 5.841) *
	Primary	3.646 (1.758, 5.534)*	1.913 (0.194, 3.632)
	Secondary	2.223 (0.225, 4.221)***	0.698 (−1.051, 2.448)
	Tertiary	Reference	
Residence	Rural	4.106 (2.743, 5.468)*	2.616 (1.242, 3.990) *
	Urban	Reference	
Diagnosis	Asthma	Reference	
	Hypertension	1.631 (−1.249, 4.511)	2.007 (−0.431, 4.445)
	Diabetes	−0.473 (−3.014, 2.067)	−0.084 (−2.241, 2.073)
	CVD	−0.388 (−3.819, 3.043)	0.865 (−2.029, 3.758)
	Others^a^	0.512 (−2.532, 3.556)	1.538 (−0.987, 4.063)
Duration of illness	≤5 years	−1.770 (−4.223, 0.6684)	−4.222 (−6.358, −2.087)*
	6–10 years	−1.572 (−4.148, 1.004)	−2.087 (−4.206, 0.031)
	≥11 years	Reference	
Comorbid diagnosis	Yes	−3.649 (−5.117, −2.181)*	1.107 (−0.352, 2.565)
	No	Reference	
Alcohol use in the past 3 months	Yes	−9.567 (−11.801, −7.332)*	−4.574 (−6.905, −2.243)*
	No	Reference	
Cigarette use in the past 3 months	Yes	−10.168 (−14.395, −5.941)*	−3.890 (−7.972, 0.192)
	No	Reference	
Khat Use in the past 3 months	Yes	−6.005 (−8.564, −3.446)*	−0.386 (−3.062, 2.291)
	No	Reference	

*p < 0.001; **p < 0.01; ***p < 0.05.

CVD, cardiovascular disorders; CMD, common mental disorders; ISI, Insomnia Severity Index; SRQ-20, Self-Reported Questionaire-20.

^a^Epilepsy, stroke, neurological, renal, and hepatologic disorders.

^†^Multiple linear regression model reported that *F*(21, 621) = 17.811, *p* < 0.001, with *R*^2^ = 0.380.

## Discussion

The global COVID-19 outbreak has wreaked havoc. Millions of lives were lost, and billions of people suffered psychologically and economically as a result. This study was used to assess the QOL of people who had chronic medical illnesses, as well as factors that were found to be significantly associated with QOL during the COVID-19 pandemic.

When the total WHOQOL-BRIEF score was compared to the sample socio-demographic characteristics, age was found to be a significant predictor, with QOL decreasing as age increased. This result was consistent with recent follow-up studies that found out older age was a risk factor for poor QOL (11, 35). This association can be explained by the fact that older age is associated with lower levels of overall health and physical function (12). QOL is projected to decline as people age, as they are more likely to suffer from many health problems (36). Another explanation could be that when people become older, the risk of COVID-19 infection increases severely (37). This suggests that during the COVID-19 pandemic, aging had a detrimental impact on QOL scores among chronic medical ill individuals.

In our study, the QOL scores of female patients were significantly lower than that of male patients, indicating that their QOL was significantly worse. Similar associations have been discovered in earlier studies, which supports our conclusion (12, 35). During the COVID-19 outbreak, women were shown to be more vulnerable to a variety of psychological problems (such as anxiety and depression) as compared to men (38, 39). Moreover, it is a known fact that females are more likely to have a lower income, more hurdles to healthcare access, and more domestic task obligations. All of these reasons may have contributed to the poor QOL in females.

We found out that educational status is significantly associated with QOL, i.e., lower educational attainment has better QOL scores. This is supported by a recent study, which found that QOL scores were shown to be lower in patients with a higher degree of education (8), due to a higher level of awareness and concern about COVID-19 and its negative effect on QOL. In addition, according to Nguyen et al. those with a high level of education had a higher prevalence of depression during the pandemic, resulting in a stress burden that adversely affects their health-related QOL (40). On the contrary, previous studies also found that, with higher levels of education, there was a general increment in QOL scores (18, 41). Higher educational attainment is often associated with greater career prospects and higher earnings, hence improving an individual’s QOL (41). However, uneducated participants claimed that their QOL was barely adequate in terms of thinking capacity, perceived physical safety and security, and vitality (41). Another rationale is that highly educated people may have a lot of wants and requirements in their daily lives, which may be jeopardized by the COVID-19 pandemic.

In our study, rural resident participants had higher QOL scores as compared to their counterparts. This is in line with previous study conducted by Hawlader et al. in Bangladesh (18). In urban areas, high population density and pollution levels may also have a negative impact on subjective QOL (42).

Duration of illness is another significant predictor of QOL in our study. Those patients with ≤5 years duration of illness have lower QOL score as compared to those with ≥11 years. This may be because of the fact that QOL can improve over time as patients adjust to chronic illness, symptoms stabilize, and more effective treatment alternatives become available over the course of long-term illness (43–45).

Our study showed that the QOL score was found to be lower among alcohol users when compared to non-alcohol users. Similar findings were reported in previous studies such that alcoholism is related to a reduced QOL (46, 47). Alcohol use has been shown to rise during stressful times such as pandemics (48). Alcohol consumption has a variety of intangible negative consequences, such as suffering, loss of healthy living, and deterioration of social and familial bonding, all of which contribute to a decrease in the individual’s QOL (49). In addition, alcohol is related to an increased risk of weakening the immune system, making people more susceptible to infectious disorders such as COVID-19 (50). Individuals use alcohol to cope with COVID-19′s stressful adaptive challenges (51). Some studies have revealed that those who drink have a much lower QOL, particularly in terms of their mental health and social functioning (52, 53).

Our study also showed that participants who had CMD had a higher probability of having lower QOL scores. The findings were consistent with a prior study, which found lower QOL scores in those who reported higher levels of anxiety, depression, and stress (9). Depression and anxiety have been linked to cognitive dysfunction (54), physical distress (55), and poor social functioning (56), all of which have been associated with a reduction in patients’ QOL. Previous studies well established that there is an inverse association between QOL and mental health problems such as depression and anxiety (57). As a result, it is not surprising that having more perceived CMDs lowers the QOL of patients with chronic medical illness, as found by this study.

This study found a statistically significant negative association between the insomnia severity index scale and the QOL scale. Similarly, recent studies indicated that impaired QOL was found to be independently associated with lower sleep quality and insomnia (58, 59). Insomnia’s impact on QOL could be due to physical or mental health comorbidities, medications, and/or a variety of psychosocial issues, or it could be a symptom of a primary disease. Furthermore, insomnia causes significant impairments in occupational and social functioning, as evidenced by decreased productivity of work, recurrent absenteeism, decreased cognition and mood, and increased physical and psychological morbidity (60). These directly or indirectly affects QOL of individuals.

This study has some limitations. First, we recognize that a cross-sectional study could not detect the continuing impact of the COVID-19 pandemic on different dimensions of QOL; thus, future research could be done using data based on a longitudinal design. Second, we did not use preferred tools to assess QOL such as SF-6D that was primarily designed to measure QOL in clinical populations. Third, because only participants from south Ethiopia were included in the study, the results cannot be applied to all Ethiopians who have chronic NCDs. These results need to be verified by additional research using a larger sample size and perhaps even a qualitative assessment. Additionally, the use of the non-probability consecutive sampling method could be viewed as a limitation. Fourth, there is a potential for social desirability bias. For instance, when data were collected using an interviewer-administered method, participants may have over- or underreported their responses for a variety of reasons.

## Conclusion

In general, increase in age, higher SRQ-20 score (having CMD), increased ISI score (insomnia), being female, shorter duration of illness (<5 years), and alcohol use have a significant negative association with high QOL in patients with chronic medical condition during COVID-19 pandemic, whereas being illiterate and living in rural residence have a positive association with high QOL. Overall, the findings confirmed that the COVID-19 pandemic had a negative impact on an individual’s QOL in a variety of ways. Thus, this study suggests that addressing insomnia, co-morbidities of mental disorders, and alcohol use has the potential effect to improve the QOL of patients with chronic medical illnesses.

## Data availability statement

The original contributions presented in this study are included in the article/Supplementary material, further inquiries can be directed to the corresponding author/s.

## Ethics statement

The studies involving human participants were reviewed and approved by Hawassa University, College of Medicine and Health Sciences, Institutional Review Board (IRB) with reference number: IRB/076/13. The participants provided their written informed consent to participate in this study.

## Author contributions

MA, BD, AG, and SH participated in the conception, designed the study, and were involved in the data collection. MA, BD, and SD performed the analysis of the study. MA and SD prepared the manuscript for publication. BD, AG, SH, and SD critically reviewed the manuscript. All authors read and approved the final manuscript.
