# Supplementary material for: Quality of life among patients with chronic non-communicable diseases during COVID-19 pandemic in Southern Ethiopia: A cross-sectional analytical study
Source: Front Psychiatry. 2022 Sep 21;13:855016. doi: 10.3389/fpsyt.2022.855016 (PMC9532738; doi:10.3389/fpsyt.2022.855016)
Supplement: Supplementary file 1 [file Table_1.DOCX]

**Supplementary File_1**

The list of items adapted to assess QoL in the context of the COVID-19 pandemic.

| S.No. | Items before Adaptation on WHOQOL-BREF | Items after Adaptation |
| --- | --- | --- |
| 1 | How would you rate your quality of life? | How would you rate the impacts of COVID-19 pandemic on your quality of life? |
| 2 | How satisfied are you with your health? | How would you rate impacts of COVID-19 pandemic on your general health? |
| 3 | How safe do you feel in your daily life? | How would you rate the impacts of COVID-19 pandemic on your feelings of being safe in your daily life? |
| 4 | How healthy is your physical environment? | How would you rate the impacts of COVID-19 pandemic on your physical environment? |
| 5 | How available to you is the information that you need in your day-to-day life? | Keeping in view the impacts of COVID-19 pandemic, how available to you was the information that you needed in your daily life? |
| 6 | Have you enough money to meet your needs? | How would you rate the impacts of COVID-19 pandemic on your income? |
| 7 | How satisfied are you with your access to health services? | How would you rate the impacts of COVID-19 pandemic on your access to health services? |
| 8 | How satisfied are you with your personal relationships? | How would you rate the impacts of COVID-19 pandemic in maintaining relationship with your friends? |
| 9 | How satisfied are you with your personal relationships? | How would you rate the impacts of COVID-19 pandemic in maintaining relationship with your family? |
| 10 | How satisfied are you with the support you get from your friends? | Keeping in view the impacts of COVID-19 pandemic, ‘how satisfied were you with the support you get from your friends?’ |
| 11 | To what extent does faith give you comfort in daily life? | To what extent does faith give you comfort to deal with hard time of COVID-19 pandemic? |
| 12 | To what extent does any connection to a spiritual hard time? | How would you rate the impacts of COVID-19 pandemic on your being help you to get through spiritual connections/practice? |
